# Mesenchymal Stem Cells: Are We Ready for Clinical Application in Transplantation and Tissue Regeneration?

**DOI:** 10.3389/fimmu.2013.00144

**Published:** 2013-06-11

**Authors:** Martin J. Hoogduijn, Frank J. M. F. Dor

**Affiliations:** ^1^Nephrology and Transplantation, Internal Medicine, Erasmus Medical CenterRotterdam, Netherlands; ^2^Surgery, Erasmus Medical CenterRotterdam, Netherlands

Mesenchymal stem cells (MSC) are emerging as a therapeutic option for a plethora of immunological and degenerative diseases. Preclinical research is focusing on the mechanisms of MSC-mediated immunomodulation and regeneration. This Research Topic shines light on mechanistic and regulatory aspects that are of importance for understanding the full potential of MSC for clinical use in transplantation and tissue regeneration.

A first consideration to make when studying MSC that is addressed in this Research Topic is the source of the cells. MSC are present in bone marrow and adipose tissue, amongst other tissues, and can be used in an autologous or allogeneic way. A commercially available allogeneic “off the shelf” cell product as described by Vaes et al. (2012) has the advantage that it is thoroughly characterized and can be produced at a relatively low cost, although for particular applications in transplantation autologous cell usage may be preferable because of the expression of HLA class I on MSC and the potential risk of sensitization upon the use of allogeneic MSC.

Functionally, MSC are influenced by environmental factors. Leijs et al. (2012) demonstrated that synovial fluid of arthritic patients modulates the expression of genes with immunomodulatory function in MSC. Also, toll-like receptor activation by pathogen-derived components or danger signals produced upon tissue injury can modulate MSC toward either an anti-inflammatory or a pro-inflammatory phenotype and alter their function (Delarosa et al., 2012). The local milieu appears therefore crucial in the therapeutic effect of MSC.

It becomes more and more clear that multiple mechanisms are responsible for the immunomodulatory effect of MSC. Different mechanisms target a variety of facets of immune cell functioning. For instance, as shown by Zinocker and Vaage (2012). The inhibition of T cell proliferation by rat MSC is dependent on nitric oxide, whereas cytokine production is modulated by the production of prostaglandin E2 by MSC. Interest is rising in the effect of MSC on B cells, as B cells are increasingly held responsible for transplant rejection. MSC can inhibit immunoglobulin production by B cells and may induce regulatory B cells, although the conditions under which this takes place are not clear yet, as lined out by Franquesa et al. (2012). More is known about the effect of MSC on regulatory T cells. Engela et al. (2012) reviewed studies that demonstrate the induction of regulatory T cells by MSC *in vitro* and *in vivo* after infusion. Although MSC and regulatory T cells target the same cell types, there is no evidence that they impede each other's function. The induction of regulatory T cells by MSC may be crucial for the long-term effects of MSC after infusion. Eggenhofer et al. (2012) demonstrated that intravenous infusion of MSC leads to accumulation of the cells in the lungs and that they disappear within 24 h, after which they cannot be detected in other tissues. This suggests that MSC are short-lived after infusion and that their effects are transferred to other cell types.

Chen et al. (2012) reviewed the clinical potential of MSC-based regenerative therapy for chronic wounds. Studies have shown that MSC administration augments the acute inflammatory response, enhances angiogenesis, accelerates re-epithelialization, and increases wound healing, even in conditions of impaired healing such as diabetes. However, whether MSC induced cutaneous regeneration by cellular differentiation or indirectly through paracrine activity is still unknown. A better understanding of the mechanism of action is needed to develop more efficient treatment strategies. Also, further investigation into delivery methods specifically designed for the delivery of progenitor cells to chronic wounds is necessary to maximize the regenerative properties of MSC-based cell therapy.

Different studies have reported beneficial effects of human MSC on repair of ischemia-reperfusion and other acute kidney injury, as discussed by de Vries et al. (2012). The therapeutic potential of human MSC was studied in immunodeficient NOD-SCID mice after cisplatin-induced acute renal failure; MSC reduced renal cell apoptosis and increased proliferation. MSC also preserved the integrity of the tubular epithelium and peritubular vessels, and prolonged survival.

Mechanistically, there is growing evidence that the process of transdifferentiation is unlikely to be relevant to renal repair *in vivo*. The primary means of these cells most likely involve paracrine and endocrine effects, including mitogenic, anti-apoptotic, anti-inflammatory, antifibrotic, and angiogenic influences. The factors that mediate the paracrine effects are obviously of great interest. Several factors that are abundant in MSC-conditioned medium have been mentioned, including microvesicles released from MSC may account for this paracrine mechanism.

Seifert et al. (2012) warn that treatment with both donor- and recipient-specific MSC in a preclinical kidney transplantation model surprisingly resulted in enhanced humoral immune responses. Signs of inflammation and rejection were generally enhanced in both MSC-treated groups compared to PBS control groups. Additionally, pre-treatment with donor-specific MSC significantly enhanced the level of donor-specific antibody formation when compared with PBS- or recipient MSC-treated groups. Pre-treatment with both MSC types resulted in a higher degree of kidney cortex tissue damage and elevated creatinine levels at the time point of rejection. Thus, MSC pre-sensitization in this model impaired the renal allograft outcome. In liver disease, it is thought that MSC act differently according to their pleiotropic spectrum of action, depending on the etiology, and pathophysiology of the specific liver disease. Thus, the anti-inflammatory, anti-apoptotic, and pro-proliferative features of MSC might be favorable in cases of chronic inflammatory liver diseases. Additionally, functional tissue replacement is warranted in cases of massive tissue loss in order to provide sufficient metabolic capacity, such as in acute liver failure and extended liver resections, as discussed by Christ and Stock (2012). Therefore, understanding the impact of MSC both on the molecular and cellular level and their interactions with the host liver tissue in a microenvironment created by the diseased liver, is important for the development of effective MSC therapy. Another interesting option is to administer MSC in combination with human hepatocytes; to support hepatocyte function, but moreover to minimize short-term rejection of the hepatocyte transplant. This would facilitate bridging the patient to liver transplantation, or help the patient through the critical phase of acute liver failure until the native liver recovers. This setting would enable allogeneic hepatocyte transplantation avoiding long-term immunosuppression and its adverse effects.

The translation of a cell-based therapy from bench to bedside is challenging under a regulatory framework involving multiple responsible authorities, EU members, and continents. Regulatory centralization has been introduced in the EU since 2009 but the remaining national procedures can be quite heterogeneous. As pointed out by Ancans, European Medical Agency (EMA), and National Competent Authorities (NCA) guidance documents, initiatives, and interaction platforms are available to make the regulatory framework more understandable and accessible for investigators both in the public and private sectors (Ancans, 2012). Good understanding of the regulatory framework is essential for product development; initial steps will determine the marketing authorization application stage. Therefore, early communications between researchers/clinicians and the NCAs and the EMA are advised. Resulting strategy improvements may facilitate MSC-based medicine development and authorization in the European Union.

The tremendous therapeutic potential of MSC has once again been highlighted in this Research Topic. MSC can alleviate alloreactivity, but also reduce inflammatory responses involved in ischemia/reperfusion injury. We are encouraged by the results of the first clinical trials, however, as we have seen in this topic, there are still several questions to be resolved before MSC can be broadly applied in transplantation. The knowledge obtained from this Research Topic may aid to further development of MSC-based therapies in transplantation and tissue regeneration.

## References

[B1] Ancans J. (2012). Cell therapy medicinal product regulatory framework in Europe and its application for MSC-based therapy development. Front. Immunol. 3:253 10.3389/fimmu.2012.00253 22912639 PMC3418507

[B2] Chen J. S. Wong V. W. Gurtner G. C. (2012). Therapeutic potential of bone marrow-derived mesenchymal stem cells for cutaneous wound healing. Front. Immunol. 3:192 10.3389/fimmu.2012.00192 22787462 PMC3392692

[B3] Christ B. Stock P. (2012). Mesenchymal stem cell-derived hepatocytes for functional liver replacement. Front. Immunol. 3:168 10.3389/fimmu.2012.00168 22737154 PMC3381218

[B4] de Vries D. K. Schaapherder A. F. Reinders M. E. (2012). Mesenchymal stromal cells in renal ischemia/reperfusion injury. Front. Immunol. 3:162 10.3389/fimmu.2012.00162 22783252 PMC3387652

[B5] Delarosa O. Dalemans W. Lombardo E. (2012). Toll-like receptors as modulators of mesenchymal stem cells. Front. Immunol. 3:182 10.3389/fimmu.2012.00182 22783256 PMC3387651

[B6] Eggenhofer E. Benseler V. Kroemer A. Popp F. C. Geissler E. K. Schlitt H. J. (2012). Mesenchymal stem cells are short-lived and do not migrate beyond the lungs after intravenous infusion. Front. Immunol. 3:297 10.3389/fimmu.2012.00297 23056000 PMC3458305

[B7] Engela A. U. Baan C. C. Dor F. J. Weimar W. Hoogduijn M. J. (2012). On the interactions between mesenchymal stem cells and regulatory T cells for immunomodulation in transplantation. Front. Immunol. 3:126 10.3389/fimmu.2012.00126 22629256 PMC3355477

[B8] Franquesa M. Hoogduijn M. J. Bestard O. Grinyo J. M. (2012). Immunomodulatory effect of mesenchymal stem cells on B cells. Front. Immunol. 3:212 10.3389/fimmu.2012.00212 22833744 PMC3400888

[B9] Leijs M. J. Van Buul G. M. Lubberts E. Bos P. K. Verhaar J. A. Hoogduijn M. J. (2012). Effect of arthritic synovial fluids on the expression of immunomodulatory factors by mesenchymal stem cells: an explorative in vitro study. Front. Immunol. 3:231 10.3389/fimmu.2012.00231 22876244 PMC3410447

[B10] Seifert M. Stolk M. Polenz D. Volk H. D. (2012). Detrimental effects of rat mesenchymal stromal cell pre-treatment in a model of acute kidney rejection. Front. Immunol. 3:202 10.3389/fimmu.2012.00202 22826709 PMC3398550

[B11] Vaes B. Van't Hof W. Deans R. Pinxteren J. (2012). Application of multistem(R) allogeneic cells for immunomodulatory therapy: clinical progress and pre-clinical challenges in prophylaxis for graft versus host disease. Front. Immunol. 3:345 10.3389/fimmu.2012.00345 23205020 PMC3506828

[B12] Zinocker S. Vaage J. T. (2012). Rat mesenchymal stromal cells inhibit T cell proliferation but not cytokine production through inducible nitric oxide synthase. Front. Immunol. 3:62 10.3389/fimmu.2012.00355 22566943 PMC3341954

